# Perceived value and barriers of nursing specialty certifications among clinical nurses in Saudi Arabia: a cross-sectional study

**DOI:** 10.3389/fmed.2025.1528856

**Published:** 2025-02-25

**Authors:** Alawiah T. AlSadah, Ahmad E. Aboshaiqah, Naif H. Alanazi

**Affiliations:** ^1^Nursing Department, King Saud University Medical City, Riyadh, Saudi Arabia; ^2^College of Nursing, King Saud University, Riyadh, Saudi Arabia

**Keywords:** barriers of certification, clinical nursesnurse clinicians, nurse specialists, nursing specialty certification, Saudi Arabia, value of certification

## Abstract

**Introduction:**

Specialty nursing certifications reflect nurse’s knowledge and competence in certain areas. Obtaining certification allows them to advance their careers and enhance patient care standards as their role and scope of responsibility expands. This study aimed to understand how nurses view specialty certification and related challenges in three university hospitals in Riyadh, Saudi Arabia.

**Methods:**

A cross-sectional, correlational design was used. A survey questionnaire (Perceived Value of Certification-12 Tool) was sent through email, and it had one question for each barrier.

**Results:**

The majority of participants valued certification to verify specialist knowledge (93.9%), enhance achievement (82.8%), and increase competence (79.1%). Certification was associated extrinsically with hiring preference (54.7%), recognition from colleagues (52.3%), and professional autonomy (51.7%). The primary barriers were lack of institutional reward (72.7%) and examination cost (64.8%), with not passing earlier being the least mentioned barrier (2%). The biggest obstacles faced by clinical nurses in obtaining certification include limited institutional rewards because their organization did offer incentives, such as promotions or salary bumps. Age and hospital significantly predicted clinical nurses’ intrinsic value of certification, while age, gender, nationality, and hospital significantly predicted their extrinsic value. Age and gender significantly influenced their overall perceived value of certification.

**Discussion:**

Overall, nurses in Riyadh recognize the value of certification. However, focused interventions must be implemented to overcome identified challenges and amplify the professional benefits of certification. Recommended measures include implementing tailored support initiatives and financial aid programs to address these barriers. A collaborative approach should be developed to provide support and actively promote the pursuit of nursing certification. Future studies should adopt longitudinal and qualitative designs to evaluate the impact of focused strategies.

## 1 Introduction

Specialty nursing certifications play an important role in improving healthcare and advancing the nursing field. These certifications show the level of commitment of nurses to become skilled in a specific area. Every day, healthcare evolves and patients’ needs increases, so having specialized certificates becomes more important than before. These certifications not only show the level of nurse’s experience but also their dedication to keep up with new practices. Certified nurses are often satisfied in their work and more confident in their abilities than non-certified ones. Certification increases not only their acknowledgements and accomplishments but also their satisfaction and confidence in their abilities. Furthermore, other nurses, employers, and patients perceive certified nurses as more knowledgeable and competent than those who are not certified. Certification may encourage them to deliver improved patient care. Although these certifications have many benefits, not all nurses can obtain them because of different challenges. This study will examine how nurses at three university hospitals perceive specialty certifications and identify the associated barriers.

Nursing specialty certification is an essential aspect of professional development that enhances nurse’s knowledge, skills, and competence in a particular field. Specialty certifications are associated with improved patient outcomes, enhanced professional credibility, and job satisfaction (1). Furthermore, this study holds importance for nurse administrators and policymakers because identifying barriers can help develop strategies to encourage nurses to pursue certifications to enhance overall staff competence and job satisfaction (2). Healthcare in Saudi Arabia undergoes significant development, so understanding how specialty certifications fit into the evolving landscape is essential to ensure that the nursing workforce is well-prepared to meet current and future healthcare demands (3).

Certified nurses are often viewed as more competent and skilled in their areas of specialization than non-certified ones, leading to high levels of trust among patients and colleagues (1). Dill et al. (2) found that certification fosters a sense of personal accomplishment and professional growth among nurses, thereby increasing their job satisfaction and retention. George (4) explored the perceptions of nurses regarding specialty certification and found that certified nurses felt confident in making clinical decisions and that their patients were more likely to report satisfaction with care. Nursing certifications are often linked to career advancement opportunities and high wages, making them a desirable option for nurses who wish to progress in their careers (5). Despite the recognized benefits, several barriers prevent nurses from pursuing specialty certifications. According to Cao et al. (6), primary obstacles include time constraints, financial costs, and lack of institutional support. In the study of Alluhidan et al. (7) on nurses in Saudi Arabia, work-related stress and burnout were identified as significant factors that hinder nurses from pursuing further qualifications. Nurses reported that their heavy workload and long shifts left little time or energy for studying and preparing for examinations.

Another prominent barrier is the lack of awareness and understanding of the value of certifications. Hinojosa (8) found that many nurses were not fully aware of the benefits of certifications or were unsure how these qualifications would translate into tangible improvements in their professional careers. This lack of knowledge often results in reluctance to invest the time and effort needed to obtain certification (9).

Cultural factors play a role in shaping the barriers to specialty certifications in Saudi Arabia. Feliciano (10) highlighted the predominance of a Clan culture, which emphasizes collaboration and close relationships, in the organizational culture within Saudi nursing schools. Although this culture fosters a supportive environment, it may inadvertently reduce the emphasis on individual achievement and professional development, thus acting as a barrier to nurses seeking certifications.

Institutional and organizational support plays a crucial role in facilitating or hindering the pursuit of nursing specialty certifications. Wei et al. (11) emphasized that supportive leadership and financial assistance, such as tuition reimbursement programs, can significantly encourage nurses to pursue specialty certifications. In teaching hospitals, where the focus is on the development and training of healthcare professionals, such support is important (12).

In Saudi Arabia, Salma and Waelli (13) found that hospitals offering financial incentives and recognition programs witnessed a high rate of nurses pursuing certifications. These programs, when combined with mentorship and career development plans, can help mitigate many of the barriers identified in the literature. The perceived value of nursing specialty certifications in teaching hospitals is clear, and it has numerous benefits for nurses and their patients (14). However, significant barriers, including work-related stress, financial costs, lack of awareness, and cultural factors, prevent many nurses from pursuing these certifications (15). Addressing these challenges through institutional support, financial assistance, and education programs can encourage nurses to seek certification, ultimately leading to improved patient outcomes and enhanced professional development (16).

## 2 Materials and methods

### 2.1 Design and setting

The study utilized a descriptive quantitative exploratory cross-sectional design to examine the perceived value of certification among nurses in three prominent hospitals in Riyadh, namely, King Khalid University Hospital (KKUH), King Abdulaziz University Hospital (KAUH), and Dental University Hospital (DUH), which are all under King Saud University Medical City (KSUMC).

KKUH is one of the largest teaching hospitals in Riyadh and offers a wide range of specialties. The hospital has a capacity ranging from 800 to 1,200 beds and accommodates a diverse patient population. Operation rooms range from 12 to 32, enabling a variety of surgical procedures. KKUH also follows strict policies regarding nursing certifications to encourage professional development among its nursing staff. KAUH is a smaller hospital with approximately 400 beds. It offers specialties in general medicine, surgery, and pediatric care. The nurse-to-patient ratio is about 1:5, and the hospital sees around 350,000 patients per year. The last hospital is DUH, which specializes in dental and oral healthcare and has around 100 beds and a nurse-to-patient ratio of about 1:2 in clinical settings. It treats approximately 150,000 patients annually.

### 2.2 Sampling and sample size

The study focused on a population of 2,350 nurses working at KKUH, KAUH, and DUH. A total of 334 nurses were randomly selected to ensure accurate results, with a 95% confidence level and a 0.05 margin of error. The inclusion criterion was nurses with more than 2 years of experience, including managers, specialty managers, and leaders. Nurses with less than 2 years of experience and patient care technicians were excluded.

### 2.3 Instrumentation and data collection

The Perceived Value of Certification Tool-Revised (16) is a 12-item self-report instrument designed by the Competency and Credentialing Institute to elicit nurses’ perceptions of the intrinsic and extrinsic value of specialty nursing certification. Respondents are asked to report the extent to which they agree or disagree with each statement about how specialty certification benefits nurses by using a 4-point Likert scale. Scores range from strongly disagree (1) to strongly agree (4). The 12-item PVCT can be divided into two subscales: intrinsic value (6 items) and extrinsic value (6 items). Intrinsic value statements focus on benefits and motivators related to obtaining a certification that are internal to the individual (e.g., “nurses who have obtained certification feel a strong sense of accomplishment”). Extrinsic value statements involve benefits and motivators related to obtaining a certification that are external to the individual or defined by others (e.g., “certified nurses generally make more money than noncertified nurses”). The 12-item PVCT exhibits a stable factor structure, measurement model fit (RMSEA = 0.07, CFI = 0.97), and adequate reliability (intrinsic *α* = 0.74–0.83; extrinsic *α* = 0.83–0.86) across various nursing credentialing organizations, certified nurses, and non-certified nurses, indicating its construct and concurrent validity (16, 31).

A total of 334 nurses were selected using simple random sampling. Emails were first sent to head nurses, who then randomly shared the survey with their staff. The questionnaire was distributed electronically, and all participants willingly completed and returned it.

### 2.4 Ethical considerations

Ethical approval was obtained from the Institutional Review Board (IRB) in King Saud University Medical City prior to the conduct of the study. Data obtained from the participants were anonymous and confidential. An implied consent process was implemented, and participants were informed about the goal and nature of the study.

### 2.5 Data analysis

Statistical analysis was conducted using SPSS version 28 (IBM Co., Armonk, NY, United States). Quantitative data were presented as mean and standard deviation (SD) and analyzed using independent Student’s t-test for the comparison of two groups or one-way ANOVA (F) for the comparison of more than two groups. Categorical data were presented as frequency and percentage (%). Linear regression analysis was performed to assess different factors associated with the studied scores. A two-tailed *p* value of <0.05 was considered statistically significant.

## 3 Results

A total of 344 participants (105 males and 239 females) were included in this study (Table 1). Among which, 49.4% were 23 to 35 years old, and 33.7% were 36 to 45 years old. More than half of the participants (52.9%) were Filipinos, 20.3% were Saudis, and 17.2% were Indians. Regarding educational level, 74.1% had bachelor’s degree, 14.2% had master’s degree and above, and 11.6% had Diploma degree. Out of the included respondents, 21.8% were assuming a leadership role (administrator, Educator, Manager, Director). Most participants (77.3%) were working at KKUH, while 13.1 and 9.6% worked at KAUH and DUH, respectively.

**Table 1:** Participants’ demographics (*N* = 334).

Demographics	*N*	%
Age (years)		
23–35	170	49.4
36–45	116	33.7
46–55	51	14.8
56–60	7	2
Gender		
Male	105	30.5
Female	239	69.5
Nationality		
Saudi	70	20.3
Filipino	182	52.9
Indian	59	17.2
Jordanian	28	8.1
Other	5	1.5
Educational Level		
Bachelor’s degree	255	74.1
Diploma degree	40	11.6
Masters degree and above	49	14.2
Occupying a leadership role (administrator, educator, manager, director)	75	21.8
Hospital		
KKUH	266	77.3
KAUH	45	13.1
DUH	33	9.6

Intrinsic value of certification was assessed by evaluating levels of participants’ agreement upon some statements (Table 2); of which, the highest level of agreement was observed for the statement that certification validated specialized clinical knowledge, which had a total agreement rate of 93.9%. The second was the statement that nurses with certification felt a strong sense of accomplishment (82.8%). The statement with the least level of agreement was the opinion that certified nurses were more competent than nurses who were not (79.1%), which had a mean intrinsic value score of 18.42 ± 3.46.

**Table 2:** Intrinsic and extrinsic values survey.

Intrinsic value survey	Strongly disagree	Disagree	Agree	Strongly agree
Certification validates specialized clinical knowledge	15 (4.4%)	6 (1.7%)	186 (54.1%)	137 (39.8%)
In my field of practice, I find that certified nurses are more competent than nurses who are not certified	17 (4.9%)	55 (16%)	158 (45.9%)	114 (33.1%)
Nurses that have obtained certification feel a strong sense of accomplishment	22 (6.4%)	37 (10.8%)	184 (53.5%)	101 (29.4%)
Certified nurses have more confidence in their abilities than non-certified nurses	24 (7%)	59 (17.2%)	171 (49.7%)	90 (26.2%)
Obtaining certification is one of the most challenging aspects of the nursing profession	29 (8.4%)	57 (16.6%)	166 (48.3%)	92 (26.7%)
Obtaining certification shows that a nurse is committed to the nursing profession	25 (7.3%)	48 (14%)	135 (39.2%)	136 (39.5%)
Score	Mean	SD	Minimum	Maximum
	18.42	3.46	6	24
Extrinsic value survey	Strongly disagree	Disagree	Agree	Strongly agree
Nurses that have obtained certification receive greater professional recognition from peers than non-certified nurses	53 (15.4%)	111 (32.3%)	120 (34.9%)	60 (17.4%)
Other medical professionals are more likely to listen to a certified nurse than a non-certified nurse	65 (18.9%)	143 (41.6%)	109 (31.7%)	27 (7.8%)
Consumers are more confident in certified nurses than non-certified nurses	115 (33.4%)	101 (29.4%)	104 (30.2%)	24 (7%)
Certified nurses are given more professional autonomy than non-certified nurses	82 (23.8%)	84 (24.4%)	136 (39.5%)	42 (12.2%)
Employers tend to favor hiring certified nurses over non-certified nurses	75 (21.8%)	81 (23.5%)	148 (43%)	40 (11.6%)
Certified nurses generally make more money than non-certified nurses	124 (36%)	91 (26.5%)	103 (29.9%)	26 (7.6%)
Score	Mean	SD	Minimum	Maximum
	13.87	3.98	6	24
Total value score	Mean	SD	Minimum	Maximum
Total score	32.29	5.84	12	48

For extrinsic value statements (Table 2), most participants agreed that employers tended to favor hiring certified nurses over non-certified nurses, with the highest level of agreement of 54.7%. About 52.3% agreed that nurses who obtained certification received greater professional recognition from peers than non-certified nurses, and 54.7% agreed that certified nurses were given more professional autonomy. The mean extrinsic value score was 13.87 ± 3.98.

Adjusting for all variables in multiple regression model revealed that age and workplace were significantly associated with intrinsic value score (Table 3). Participants in the age group of 46–55 years had a higher score than those in the age group of 23–35 years [(95%CI) of 1.16 (0.01 to 2.31), *p* = 0.049]. In terms of workplace, participants working at KAUH and DUH had lower scores than those working at KKUH [(95%CI) of −1.39 (−2.5 to −0.27), *p* = 0.015] and [−1.44 (−2.75 to −0.13), *p* = 0.031].

**Table 3:** Multiple linear regression analysis for factors associated with intrinsic value score.

	Coefficient	95%CI	*P* value
Age (years)			
23–35	Ref		
36–45	0.63	−0.21 to 1.46	0.141
46–55	1.16	0.01 to 2.31	0.049*
56–60	1.46	−1.22 to 4.13	0.285
Gender			
Male	Ref		
Female	−0.07	−0.9 to 0.77	0.871
Nationality			
Saudi	Ref		
Filipino	−0.15	−1.17 to 0.88	0.778
Indian	−0.21	−1.59 to 1.18	0.768
Jordanian	−0.53	−2.07 to 1	0.494
Other	−0.97	−4.22 to 2.27	0.556
Educational level			
Bachelor’s degree	Ref		
Diploma degree	−1.15	−2.5 to 0.21	0.096
Masters degree and above	1.09	−0.2 to 2.38	0.097
Occupying a leadership role			
No	Ref		
Yes	0.4	−0.7 to 1.5	0.477
Hospital			
KKUH	Ref		
KAUH	−1.39	−2.5 to −0.27	0.015*
DUH	−1.44	−2.75 to −0.13	0.031*

CI: confidence interval, *Bold value: statistically significant as *p* value <0.05.

Age, gender, nationality, and workplace were significantly associated with the extrinsic value score (Table 4). Participants in the age group of 46–55 years had higher scores than those in the age group of 23–35 years [(95%CI) of 1.79 (0.54 to 3.04), *p* = 0.005]. Female participants had significantly higher scores than males [(95%CI) = 1.65 (0.75 to 2.56), *p* < 0.001]. Jordanian participants had significantly lower scores than Saudis [(95%CI) = −1.87 (−3.54 to −0.2), *p* = 0.028]. In terms of workplace, participants working at KAUH and DUH had higher scores than those working at KKUH [(95%CI) of, respectively, [2.57 (1.36 to 3.78), *p* < 0.001] and [1.78 (0.35 to 3.2), *p* = 0.014]].

**Table 4:** Multiple linear regression analysis for factors associated with extrinsic value score.

	Coefficient	95%CI	*P* value
Age (years)			
23–35	Ref		
36–45	0.05	−0.85 to 0.96	0.907
46–55	1.79	0.54 to 3.04	0.005*
56–60	2.27	−0.63 to 5.17	0.125
Gender			
Male	Ref		
Female	1.65	0.75 to 2.56	<0.001*
Nationality			
Saudi	Ref		
Filipino	−0.78	−1.9 to 0.33	0.167
Indian	−0.24	−1.74 to 1.26	0.754
Jordanian	−1.87	−3.54 to −0.2	0.028*
Other	0.07	−3.45 to 3.59	0.969
Educational Level			
Bachelor’s degree	Ref		
Diploma degree	−0.21	−1.68 to 1.25	0.774
Masters Degree and above	0.61	−0.79 to 2.01	0.395
Occupying a leadership role			
No	Ref		
Yes	0.88	−0.32 to 2.07	0.149
Hospital			
KKUH	Ref		
KAUH	2.57	1.36 to 3.78	<0.001*
DUH	1.78	0.35 to 3.2	0.014*

CI: confidence interval, *Bold value: statistically significant as *p* value <0.05.

Age and gender were significantly associated with the total value score (Table 5). Participants in the age group of 46–55 years had higher scores than those in the age group of 23–35 years [(95%CI) of 2.95 (1.02 to 4.87), *p* = 0.003]. Female participants had significantly higher scores than males [(95%CI) of 1.59 (0.19 to 2.98), *p* = 0.026].

**Table 5:** Multiple linear regression analysis for factors associated with total value score.

	Coefficient	95%CI	*P* value
Age (years)			
23–35	Ref		
36–45	0.68	−0.71 to 2.08	0.337
46–55	2.95	1.02 to 4.87	0.003*
56–60	3.73	−0.73 to 8.19	0.101
Gender			
Male	Ref		
Female	1.59	0.19 to 2.98	0.026*
Nationality			
Saudi	Ref		
Filipino	−0.93	−2.64 to 0.78	0.285
Indian	−0.45	−2.76 to 1.86	0.703
Jordanian	−2.4	−4.97 to 0.16	0.066
Other	−0.91	−6.32 to 4.51	0.742
Educational level			
Bachelor’s degree	Ref		
Diploma degree	−1.36	−3.62 to 0.89	0.236
Masters degree and above	1.7	−0.45 to 3.85	0.122
Occupying a leadership role			
No	Ref		
Yes	1.27	−0.56 to 3.11	0.172
Hospital			
KKUH	Ref		
KAUH	1.18	−0.68 to 3.04	0.212
DUH	0.33	−1.85 to 2.52	0.763

CI: Confidence interval, *Bold value: statistically significant as *p* value <0.05.

**Figure 1:**
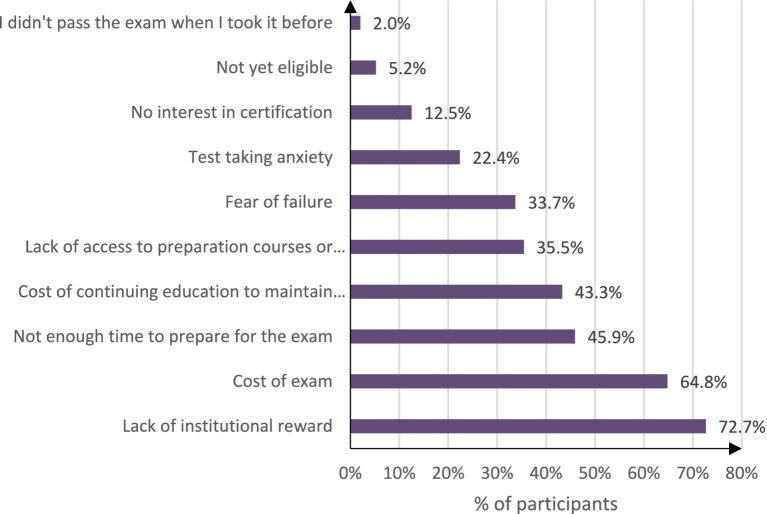
Perceived barriers of nursing specialty certifications.

The biggest obstacles faced by nurses in obtaining certifications include limited institutional rewards, with 72.7% of the participants saying that their organization did not offer incentives, such as promotions or salary bumps. The second barrier was the fees of the examination, it affected 64.8% of respondents who felt the cost is too high. Another barrier was time constraint, which had a score of 45.9%. They stated difficulty to fit studying into their work and personal lives. Access to preparation resources is another problem, with 35.5% of the respondents reported difficulty in obtaining the materials they need. Many of the participants also mentioned psychological strain, such as test anxiety (22.4%) and fear of failure (33.7%). Together, these factors make certification seem like a difficult battle for many professionals, thereby reducing their motivation and participation (see Figure 1).

## 4 Discussion

This research explored the intrinsic and extrinsic values that nurses in Saudi Arabia associate with professional nursing certifications, in addition to other important factors that may influence these perceived values. With respect to intrinsic value, the nurses overwhelmingly agreed that certification validates specialized clinical knowledge, with more than 93% of the respondents affirming the same. About 79% of the respondents also believed that certified nurses were more competent than nurses without certification. These results align with the findings of Ray et al. (17) and Walter et al. (18). Certification validates specialized clinical knowledge based on the perceptions of the interviewed nurses. Both categories of researchers explored nurses’ views from different specialties. In addition, the results of this study corroborate with previous research studies indicating that specialty certification among nurses have reported impacts on several essential aspects of the healthcare system. These aspects encompass the improvement of patient care quality (19–21), enhanced patient safety (12, 22), a reduction in patient mortality rates (19, 23), and the overall advancement of healthcare systems (12, 21).

This research also established that certification reveals nurses’ commitment to the profession (68.7%), boosts feelings of accomplishment (82.9%), and denotes higher competence and abilities compared with non-certified nurses (79%). Studies across North America and Europe have demonstrated that nurses perceive professional certification as validating specialized knowledge (17, 18), promoting professionalism (6), and enhancing internal feelings of personal growth (16) as well as external credibility and value to employers (13). The high intrinsic motivations of nurses to obtain credentials appear consistent across regions. The convergence around improved competence, commitment, and confidence conferring value to certification suggests universal social and psychological drivers across nursing cultures (24).

In terms of extrinsic values, agreement was obtained but to a lesser degree. Certification can provide professional recognition, autonomy, hiring preference from employers, and potentially high pay. The researchers found that 52.3% of the respondents believed that nurses who have obtained certification received greater professional recognition from peers than non-certified nurses. About 39.5% of the respondents believed that other medical professionals are more likely to listen to a certified nurse than a non-certified nurse, with consumers trusting the directives provided by certified nurses at 37.2%. While intrinsic drivers dominate, research shows that nurses across regions believe certification can yield professional opportunities (25) and monetary gain (2, 26) in addition to internal fulfillment (27).

The perception of variable extrinsic incentives dependent on context aligns with mixed evidence that certification impacts compensation (2, 26), authority (4, 15), or mobility (28). Integrating findings from different studies, it is clear that nurses recognize potential extrinsic benefits like better career opportunities or higher pay. However, some are uncertain whether certification will lead to concrete external/structural improvements. This aligns with the complicated evidence from Western/developed countries where research shows mixed or context-specific extrinsic impacts of certification. The internal motivations around competence, confidence and commitment are consistent universal drivers that initially motivate nurses across regions to seek certification (9). However, whether certification ultimately functions to advance nurses’ careers, authority, and compensation depends on the external realities of how it gets structurally embedded in different nursing labor force contexts.

When comparing mean scores, Saudi nurses place greater subjective value on the internal sense of achievement, validation, and confidence that comes with certification (intrinsic rewards) compared to external validations like recognition or career/financial perks (extrinsic factors). Still, they perceive both intrinsic and extrinsic benefits overall. The regression analysis found that intrinsic value showed a positive association with older nurse age (46–55 years) but lower perceptions among those at King Abdulaziz University Hospital (KAUH) and Dr. Soliman Fakeeh Hospital (DUH) versus staff at King Khalid University Hospital. For extrinsic value, higher age, female gender, and working at KAUH/DUH predicted greater scores, while Jordanian nationality correlated with lower extrinsic value. Total value scores were higher for older nurses (46–55 years) and for females. Work setting and other demographics did not emerge as significant for total value. The results of the current study align with previous research conducted in the United States, which indicated a correlation between the characteristics of nurses and their likelihood of obtaining specialty certification (29). In particular, the study by Dierkes et al. (29) revealed that older and more experienced nurses were more likely to obtain specialty certification. This tendency among nurses to pursue specialty certification may be attributed to various factors, including established eligibility criteria that necessitate a certain number of clinical practice hours, as well as the impact of full-time employment status (29). Additionally, the renewal of registration or the requirements for professional classification as a registered nurse in Saudi Arabia, as stipulated by the Saudi Commission for Health Specialties (30), may also play a vital role. In a qualitative study that focused on middle-aged nurses in the United States, findings suggested that healthcare organizations ought to foster an organizational culture that promotes specialty certifications among nursing professionals. Furthermore, the research identified key areas that are vital for assisting nurses in their efforts to obtain these certifications (11). The results of this current study that align with previous research (11, 29), are also consistent with a systematic review which revealed that nurses with specialty certifications had an average age of 49 and had more than 20 years of nursing experience (31).

### 4.1 Strengths and limitations

One of the key strengths of this study is its large sample size, which helps provide an accurate reflection of nurses’ perceptions in three major teaching hospitals in Riyadh. Additionally, the study covers the intrinsic and extrinsic aspects of certification value and offers a comprehensive understanding of nurses’ views.

However, this study has some limitations. It relied on self-reported data through questionnaires, which may introduce bias because participants might not provide entirely accurate responses. The study is also limited to three hospitals under KSUMC, which may not represent the views of nurses in other regions or types of healthcare settings in Saudi Arabia. Furthermore, while the study explored many factors influencing certification perceptions, it did not include other variables, such as department specialty or years of experience, which could also affect perceptions.

## 5 Conclusion

This study found that clinical nurses in Saudi Arabia had an overall overwhelmingly agreement that certification validated their specialized clinical knowledge. In particular, they reported strong agreement on the intrinsic value of specialty certification but displayed modest agreement on the extrinsic value of certification. The biggest obstacles faced by clinical nurses in obtaining certification include limited institutional rewards as their organization did not offer incentives, such as promotions or salary bumps. Other important factors, intrinsically (age and hospital), extrinsically (age, gender, nationality and hospital), and overall perceived value of certification (age and gender), influenced the perceived values among clinical nurses.

## 6 Recommendations

To make nursing specialty certification more valuable and address some of the common obstacles, healthcare organizations could start by offering financial support, including reimbursement of examination fees and ongoing certification costs. Establishing clear pathways for career growth that connect certification with pay raises or promotions can also make a big difference. Providing study resources, flexible scheduling, and peer-led study groups can help nurses manage time constraints. Workshops on stress management can ease test-related anxiety. Celebrating certification achievements in team meetings, newsletters, or providing awards could help nurses feel recognized and motivated. Healthcare organizations can raise the standard of care, encourage professional growth, and show nurses that their hard work truly matters by creating a supportive environment that ties certification to patient care quality.

## Data Availability

The original contributions presented in the study are included in the article/supplementary material, further inquiries can be directed to the corresponding author.
